# A population-based comparison of treatment patterns, resource utilization, and costs by cancer stage for Ontario patients with hormone receptor-positive/HER2-negative breast cancer

**DOI:** 10.1007/s10549-020-05960-4

**Published:** 2020-10-16

**Authors:** Christine Brezden-Masley, Kelly E. Fathers, Megan E. Coombes, Behin Pourmirza, Cloris Xue, Katarzyna J. Jerzak

**Affiliations:** 1 Division of Medical Oncology and Hematology, Faculty of Medicine, Mount Sinai Hospital, University of Toronto, Toronto, ON Canada; 2 Department of Medical Affairs, Hoffmann-La Roche Limited, Mississauga, ON Canada; 3 Market Access and Pricing Department, Hoffmann-La Roche Limited, Mississauga, ON Canada; 4 Division of Medical Oncology and Hematology, Faculty of Medicine, Sunnybrook Odette Cancer Center, University of Toronto, Toronto, ON Canada

**Keywords:** Cohort studies, Drug therapy, Health services research, Radiotherapy, Receptors steroid, Surgical procedures operative, Breast neoplasms

## Abstract

**Purpose:**

To update and expand on data related to treatment, resource utilization, and costs by cancer stage in Canadian patients with hormone receptor-positive (HR+)/human epidermal growth factor receptor 2-negative (HER2−) breast cancer (BC).

**Methods:**

We analyzed data for adult women diagnosed with invasive HR+/HER2− BC between 2012 and 2016 utilizing the publicly funded health care system in Ontario. Baseline characteristics, treatment received, and health care use were descriptively compared by cancer stage (I–III vs. IV). Resource use was multiplied by unit costs for publicly funded health care services to calculate costs.

**Results:**

Our study included 21,360 patients with stage I–III plus 813 with stage IV HR+/HER2− BC. Surgery was performed on 20,510 patients with stage I–III disease (96.0%), with the majority having a lumpectomy, and radiation was received by 15,934 (74.6%). Few (*n* = 1601, 7.8%) received neoadjuvant and most (*n* = 15,655, 76.3%) received adjuvant systemic treatment. Seven hundred and fifty eight patients with metastatic disease (93.2%) received systemic therapy; 542 (66.7%) received endocrine therapy. Annual per patient health care costs were three times higher in the stage IV vs. stage I–III cohort with inpatient hospital services representing nearly 40% of total costs.

**Conclusion:**

The costs associated with metastatic HR+/HER2− BC reflect a significant disease burden. Low endocrine treatment rates captured by the publicly funded system suggest guideline non-adherence or that a fair portion of Ontarian patients may be incurring out-of-pocket drug costs.

**Electronic supplementary material:**

The online version of this article (10.1007/s10549-020-05960-4) contains supplementary material, which is available to authorized users.

## Introduction

Breast cancer (BC) is a heterogenous disease that can be defined by morphologic or molecular features that predict a patient’s prognosis. One of the most clinically driven classification schemes relies on immunohistochemical measures of receptor expression to guide systemic therapy [1]. Tumors testing positive for hormone receptors (HR+) and negative for human epidermal growth factor receptor 2 (HER2−) represent the most common BC subtype; approximately 70% of cases [2]. Patients with HR+/HER2− BC have higher 5-year survival rates compared to those with triple negative or HER2 + BC. The median overall survival among patients with stage IV HR+/HER2− disease ranges from four to five years [2, 3].

Treatment of HR+/HER2− BC has primarily focused on endocrine therapy to downregulate HR signaling [2]. For stage I–III HR+ BC, this entails at least 5 years of tamoxifen or aromatase inhibition (AI; with anastrozole, exemestane, or letrozole in post-menopausal or ovarian suppressed women), with extended adjuvant therapy recommended for women at high risk of recurrence [4]. The decision to add adjuvant chemotherapy is informed by a combination of clinical and genomic risk assessment, as well as patient preference [5]. Standard surgical approaches include mastectomy or lumpectomy with lymph node sampling, typically followed by radiation [2].

Historically, the treatment of patients with metastatic HR+ BC has involved serial endocrine treatment as the preferred approach, except in the setting of visceral crisis or life threatening disease, when chemotherapy is preferred as first line therapy [2, 6, 7]. Recent evidence supports additional inhibition of cyclin-dependent kinase (CDK) 4/6, which has become standard of care in the first line setting, as well as inhibition of the PI3K/AKT/mTOR pathway, often in later lines of therapy [8, 9].

To date, population-based studies in BC have most commonly reported disease characteristics of a broad population or risk/outcome within a specific breast subtype. One real-world assessment of Ontario BC patients reported on direct healthcare costs by cancer stage [10], but there are no similar studies specific to the HR+/HER2− subtype. We sought to assess the occurrence, management, resource utilization, and costs according to stage I–III versus metastatic disease in Ontario women diagnosed with HR+/HER2− BC.

## Methods

### Study design

We conducted a retrospective, observational, population-based study to assess the treatment resource utilization and health care costs for a cohort of Ontario women diagnosed with stage I–III versus stage IV HR+/HER2− BC. The study was approved by the Ontario Cancer Research Ethics Board in 2017 and conducted in 2019 by ICES using all relevant databases under their purview (Table S1).

ICES is an independent non-profit organization that houses de-identified population-based health and social data on publicly funded services provided in Ontario. Cases are linked across databases by their unique Ontario Health Insurance Plan (OHIP) number. In Ontario, all Canadian citizens and permanent residents are eligible to receive publicly funded hospital care, most physician services, outpatient and emergency services, and, for those 65 years of age or older or on social assistance, prescription drug coverage. Supplemental drug funding is also provided by the government through special programs within the Ontario Drug Benefit (ODB) program or the New Drug Funding Program (NDFP).

Incident cases of invasive BC [11] in adult women (18–105 years old) diagnosed between Apr 1, 2012 and Mar 31, 2016 were extracted from the Ontario Cancer Registry (OCR). This timeframe was selected based on feasibility data from ICES and took into consideration availability of laboratory test results for HER2 and HR status as well as the target population of 40,000 women with breast cancer. Those diagnosed with a secondary non-BC malignancy were excluded from the analysis, as well as any incomplete/invalid records (i.e., missing age/gender). The final cohort for this sub study only included patients with a known and negative HER2 status, a known and positive HR status, and a documented cancer stage (Fig. S1). Molecular subtype was determined from synoptic pathology reports housed in the OCR. Receptor status was defined according to Cancer Care Ontario (CCO) and ASCO/CAP guidelines, with HR+ status defined as ER and/or PR expression of ≥ 1% by immunohistochemistry [12–14]. Patients were assigned to the stage I–III or stage IV cohort based on their stage at initial diagnosis; therefore, all patients in the stage IV cohort had de novo metastatic disease. Patients were followed until the earliest of the following: date of last contact with the health care system, end of OHIP eligibility, death, or end of study, which was Mar 31, 2017.

### Measures and data sources

Variables of interest for data collection included age, rurality [15], comorbidity index [16], income status [17], and various tumor characteristics. American Joint Committee on Cancer disease stage at diagnosis was reported according to the Collaborative Staging Methodology (v.1.0, 2004) which incorporates TNM information [18]. Tumor characteristics of interest were derived from the OCR and included histologic grade (reported according to the Nottingham combined scoring system) [19], laterality, pathologic tumor size, and lymph node status.

Treatment-related variables of interest included treatments received (surgery, radiation, or systemic therapy), time between diagnosis and the start of each treatment modality, and use of targeted and endocrine therapies according to stage. The authors reviewed database treatment codes and ensured queries related to systemic therapy were specific to anti-cancer therapies (including chemotherapies, endocrine and targeted therapies). Surgery dates and types were derived from the Canadian Institute for Health Information (CIHI) Discharge Abstract and Same Day Surgery databases. Rates of radiation therapy (RT) were calculated using radiation exposure data captured in OHIP, National Ambulatory Care Reporting System (NACRS), and/or the Cancer Activity Level Reporting (ALR) databases between diagnosis and study end. Rates of systemic therapy were calculated using drug exposure data from the OHIP, NACRS, ALR, NDFP, and/or ODB databases.

For patients with stage I–III disease, systemic therapy was categorized as neoadjuvant (NAT, occurring before surgery) or adjuvant (AT, occurring within 24 weeks after surgery—a broad window intended to ensure the capture of systemic therapy in case of delay following locoregional therapy). Since the majority of systemic anti-cancer therapies are reimbursed (by ODB or NDFP), dispensed, and administered by the cancer clinics, CCO’s ALR database was considered the most comprehensive provincial record of cancer regimens received.

Health resource utilization measures included number of events/uses as well as length of stay where applicable and were queried in the databases outlined in Table S1. Costs were determined by multiplying the health resource utilized by the unit cost. Unit costs for emergency room visits, day hospitalizations/surgeries, and inpatient/rehabilitation stays were sourced from CIHI and the Ontario Case Costing Initiative. Costs for biopsies, imaging, physician visits, and laboratory tests were sourced from the Ontario Ministry of Health and Long-Term Care (MOHLTC). Health service cost components were summed to calculate the total direct cost for the full period of care. To estimate annual direct health care costs per patient, total costs over the study period were divided by the period of care and the number of patients. All reported costs were inflated to 2017 Canadian dollars using the Consumer Price Index calculator [20].

For some reporting, costs were combined into themes, as follows

- Continuous care = long-term care + complex continuing care.
- Pharmaceutical (drug only) = ODB + NDFP.
- Inpatient = hospital + mental health + rehabilitation.
- Ambulatory non-cancer = emergency department + dialysis clinic visits.

Hospital outpatient cost data were derived from the MOHLTC and defined as billings involving day surgery, medical day care, or clinic care related to clinic attendance, rehabilitation services, or diagnostic tests. These costs were then linked to OHIP records using a validated algorithm [21].

### Statistical methods

Considering the descriptive nature of our study and that there were approximately 9614 new cases of BC each year among an Ontario population of approximately 13 million [22], our sample size was fixed as the number of cases identified over the four-year period of the study.

Results are reported using descriptive statistics for center (mean, median) and dispersion (SD and interquartile range [IQR]) for all continuous variables. Categorical variables were summarized using counts and percentages. In accordance with ICES policies, cells with fewer than six patients and any interrelated cells were suppressed to prevent re-identification.

## Results

### Patient characteristics

Among the 34,340 women newly diagnosed with BC and meeting the criteria for inclusion, 22,247 (64.8%) had HR+ BC. Notably, 3914 patients had an unknown histologic subtype and 74 had no reported disease stage and were, therefore, excluded from further analyses.

The mean age of women with stage I–III and stage IV HR+/HER2− BC was 62.1 (± 13.1) years and 62.8 (± 14.0), respectively. Table 1 highlights key demographic and tumor characteristics observed in the cohort of patients with stage I, II or III versus stage IV disease at diagnosis. Patients were evenly distributed among income quintiles and comorbidity data were missing for a significant proportion of patients.

**Table 1:** Baseline characteristics of Ontario patients with HR+/HER2− breast cancer (2012–2016) by stage at diagnosis

	Stage I–III (*n* = 21,360)		Stage IV (*n* = 813)	
	No.	%	No.	%
Age, years				
18–64	12,037	55.4	450	55.4
65+	9323	44.6	363	44.6
Index fiscal year				
2012	4975	23.3	195	24.0
2013	5260	24.6	217	26.7
2014	5529	25.9	218	26.8
2015	5596	26.2	183	22.5
Rurality of residence				
Missing	14^*a*^	0.0^*a*^	3^*a*^	0.1^*a*^
No	18,721	87.6	733	90.2
Yes	2625^a^	12.3^a^	77^a^	9.5^a^
Income quintile				
Missing	83	0.4	6	0.7
1-Lowest	3514	16.5	191	23.5
2	3989	18.7	168	20.7
3	4273	20.0	140	17.2
4	4630	21.7	168	20.7
5-Highest	4871	22.8	140	17.2
Charlson comorbidity index				
Missing	14,593	68.3	592	72.8
Mean ± SD				
Laterality of primary				
Right	10,550	49.4	395	48.6
Left	10,805	50.6	410	50.4
Unspecified, one-sided	3^a^	0.0^a^	3^a^	0.1^a^
Paired site	3^a^	0.0^a^	8^a^	1.0^a^
Primary tumor size				
No mass found	8^a^	0.0^a^	6^a^	0.7^a^
<1 cm	3435	16.1	9	1.1
1 to < 2 cm	7838	36.7	60	7.4
2 to < 5 cm	8092	37.9	330	40.6
5 cm or greater	1034	4.8	284	34.9
Other^b^	14^a^	0.1^a^	5^a^	0.6^a^
Unknown	69	0.3	119	14.6
Lymph node status				
Negative	13,220	61.9	17	2.1
Positive	6829	32.0	319	39.2
Unknown	1311	6.1	477	58.7
Tumor grade				
Grade 1	5,447^a^	25.5^a^	23^c^	2.8^c^
Grade 2	10,548	49.4	168	20.7
Grade 3	3888	18.2	101	12.4
No exam/unknown	1,479^c^	6.9^c^	523^a^	64.3^a^
Disease stage				
I	10,469	49.0	0	0.0
II	8229	38.5	0	0.0
III	2659	12.5	0	0.0
IV	0	0.0	813	100.0
HR status				
ER+/PR+	19,120	89.5	680	83.6
ER+/PR−	2087	9.8	126	15.5
ER−/PR+	153	0.7	7	0.9

*ER* estrogen receptor, *HR* hormone receptor, *PR* progesterone receptor

^a^Mid-point of suppressed data range, *n* = ±2

^b^Diffuse disease or Paget’s disease of the nipple with no tumor

^c^Mid-point of suppressed data range, *n* = ±4

Of the 22,173 patients included in the analysis, 1571 (7.1%) died within the timeframe of study follow-up (median 34 months [23–47], IQR:23–47). This included 1172 (5.5%) patients with stage I–III and 399 (49.1%) with stage IV disease with a median follow-up of 34 (IQR:23–47) and 24 (IQR: 14–36) months, respectively.

### Treatment

Rates of surgery and RT were lower in the stage IV cohort compared with the stage I–III cohort, although the metastatic cohort had higher systemic treatment rates (Table 2).

**Table 2:** Treatment received by Ontario patients with HR+/HER2− breast cancer (*n* = 22,173) by stage at diagnosis (2012–2017)

Treatment modality	Stage I–III (*n* = 21,360)		Stage IV (*n* = 813)	
	No.	%	No.	%
Surgery (within 1 year of diagnosis)	20,510	96.0	164	20.2
Lumpectomy^a^	15,043	70.4	85	10.5
Mastectomy^a^	5302	24.8^a^	75	9.2^b^
Lumpectomy followed by mastectomy^c^	120^b^	0.6^b^	3^b^	0.4^b^
Lymph node excision only	43^b^	0.2^b^	3^b^	0.4^b^
Systemic therapy^d^	16,733	78.3	758	93.2
Endocrine therapy	14.709	68.9	542	66.7
Aromatase inhibitor	9088	42.5	408	50.2
Tamoxifen	7498	35.1	229	28.2
Fulvestrant	73	0.3	78	9.6
Everolimus	44	0.2	40	4.9
CDK 4/6 inhibitor	39	0.2	17	2.1
Radiation therapy	15,934	74.6	475	58.4

In the group of patients with stage I–III disease receiving surgery (*n* = 20,510, 96.0%), the mean number of surgeries was 1.17 (± 0.42) (Table S2). The median number of days between diagnosis and first surgery was 36 (IQR: 25–51). Among patients treated with upfront surgery for early stage disease (*n* = 18,909, 88.5%), 14,436 (76.3%) received AT. The remaining 1601 (7.8%) received NAT starting a median of 29 days (IQR: 21–42) after diagnosis and underwent surgery a median of 140 days (IQR: 126–160) after the start of NAT. Approximately, 76.1% (*n* = 1219) of patients who were treated with NAT went on to receive AT after surgery. For all patients, AT was started within a median of 48 days (IQR: 34–65) of surgery. Patients undergoing surgery but not receiving systemic or RT totalled 1534 (7.5%).

For patients diagnosed with stage IV HR+/HER2− BC, surgery was the least common treatment modality (*n* = 164, 20.2%) compared with any RT (*n* = 475, 58.4%) and systemic therapy (*n* = 758, 93.2%). Of those undergoing surgery, 155 (94.5%) received systemic therapy and 105 (64.0%) received RT. Of those not undergoing surgery (*n* = 649), 603 (92.9%) received systemic treatment a median of 35 days (IQR: 23–62) after diagnosis, and 370 (57.0%) received RT (Table S3).

Among patients with early stage HR+/HER2− BC, 14,709 (68.9%) received endocrine therapy, with an AI (*n* = 9088, 42.5%) and/or tamoxifen (*n* = 7498, 35.1%) being the most common treatments. Similarly, in the metastatic setting, 542 (66.7%) received endocrine therapy, including AI (*n* = 408, 50.2%), tamoxifen (*n* = 229, 28.2%) , and/or fulvestrant (*n* = 78, 9.6%) (Table 2).

### Resource utilization and costs

**Fig. 1:**
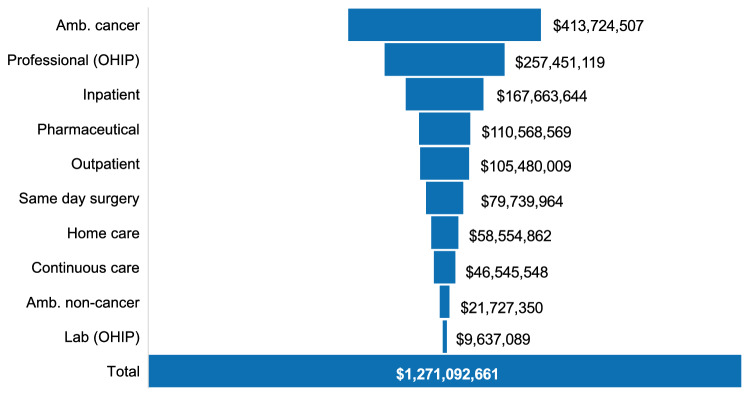
Total costs by resource type for Ontario patients with HR+/HER2− breast cancer (*n* = 22,173) across study follow-up period (2012–2017). *Amb.* ambulatory, *OHIP* Ontario Health Insurance Plan

The full HR+/HER2− cohort was responsible for total measured costs of $1,271,092,661 between April 2012 and March 2017. In terms of total measured costs by resource type, ambulatory cancer clinic visits and OHIP professional fees combined accounted for over half of the costs incurred (32.5% and 20.3%, respectively) (Fig. 1).

For patients with stage I–III HR+/HER2− BC, the average annual per patient total cost was $22,662, compared with $77,112 for patients with stage IV disease (Table 3 and S4). Costs were higher for patients with stage IV disease for all resource use categories except same day surgery, which was higher for patients with stage I–III disease. Ambulatory cancer clinic visits, hospital inpatient services, and OHIP professional fees were the primary contributors to annual costs in both subgroups.

**Table 3:** Average annual per patient cost by resource type for patients with HR+/HER2− breast cancer (*n* = 22,173) by stage at diagnosis (Ontario, 2012–2017)

	Stage I–III (*n* = 21,360)		Stage IV (*n* = 813)	
	Cost	% Of total cost	Cost	% Of total cost
Total	$22,658	100.0	$77,111	100.0
Ambulatory cancer	$7061	31.2	$15,177	19.7
Professional (OHIP)	$4617	20.4	$11,990	15.5
Inpatient	$3422	15.1	$29,484	38.2
Pharmaceutical	$1841	8.1	$4122	5.3
Outpatient	$1840	8.1	$4040	5.2
Same day surgery	$1496	6.6	$498	0.6
Home care	$1013	4.5	$4982	6.4
Continuous care	$822	3.6	$5290	6.7
Ambulatory non-cancer	$386	1.7	$1340	1.7
Lab (OHIP)	$161	0.7	$188	0.2

*OHIP* Ontario Health Insurance Plan

**Fig. 2:**
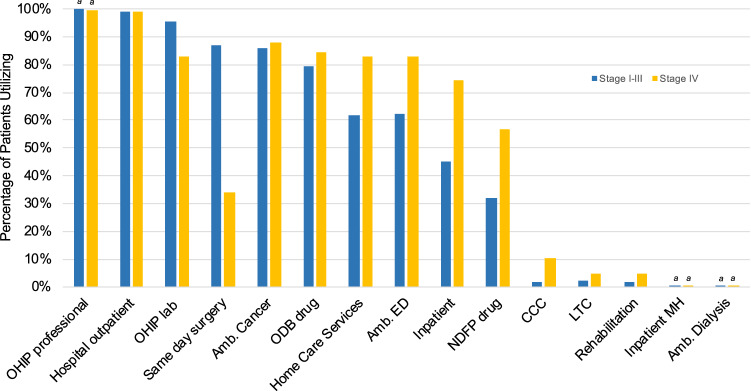
Proportion of patients with HR+/HER2− breast cancer, by stage, utilizing each health care resource (Ontario, 2012–2017). ^a^mid-point of suppressed data range; *n* = ±2. *Amb.* ambulatory, *CCC* complex continuing care, *ED* emergency, *Hosp.* hospital, *LTC* long-term care, *MH* mental health, *NDFP* New Drug Funding Program, *ODB* Ontario Drug Benefit, *OHIP* Ontario Health Insurance Plan, *Rehab.* rehabilitation

OHIP professional, hospital outpatient services, ambulatory cancer, and ODB were highly utilized and by a similar proportion of patients regardless of disease stage (Fig. 2, Table S4). Emergency, home care, and inpatient services as well as the NDFP were, in contrast, utilized by a higher proportion of patients with stage IV BC.

Of the 22,173 patients with HR+/HER2− BC, 10,176 (46%) had at least one inpatient hospital stay. Among them, patients with metastatic disease had an average number of inpatient visits three times that of patients with stage I–III disease and a substantially longer average length of stay (32.7 versus 4.6 days, respectively) (Table S4).

Patients with stage IV HR+/HER2− BC also had roughly three times the number of OHIP professional encounters and twice as many hospital outpatient visits than patients with stage I–III disease. Costs related to the use of home care service, emergency, and complex continuing care were approximately triple in patients with stage IV versus stage I–III HR+/HER2− BC (Table S4).

## Discussion

### Patient characteristics

The incidence of HR+/HER2− BC among all BC subtypes in our study was 64.8%, with 3.7% diagnosed as stage IV. These incidences are in line with those observed in a European registry analysis [23] and a US population-based case–control study [24]. Interestingly, the proportion of patients with ER positive tumors was higher in our cohort than others (99% versus 86%) [23, 24]. The majority of patients with stage I–III HR+/HER2− BC in our study had favorable prognostic features such as tumor size < 2 cm (53%), lymph node negative status (62%), and well to moderately differentiated tumor pathology (75%).

### Treatment

Patients with stage I–III HR+/HER2− BC underwent lumpectomy or mastectomy at rates of 71% and 25%, respectively, which demonstrates support for breast-conserving surgery comparable to [25] or greater than [26–29] that reported in other cohorts.

Unfortunately, because systemic therapies can be entered into the ALR database as single agents and/or combination regimens and may be entered differently with each administration, it was prohibitively difficult to discern which regimens were given and for what duration. Thus, although collected, in-depth data on systemic chemotherapy was of little value for reporting.

Considering that endocrine therapy is recommended for all patients with HR+ disease, regardless of disease characteristics, the endocrine treatment rate (68.9%) for women with early stage disease was lower than expected [30–32]. Other real-world reports have noted similar or higher rates of adjuvant endocrine therapy usage (70–86%), with some highlighting challenges related to adherence and duration of treatment [27, 33–41]. Unfortunately, an analysis of factors influencing treatment was beyond the scope of our research, but is warranted in order to optimize outcomes [42] in patients with this subtype of BC.

Systemic treatment in the de novo metastatic setting was high (93.2%); however, only 66.7% overall received endocrine therapy; a rate lower than that reported in comparable cohorts [23, 43]. Since public funding for endocrine therapy does not cover all patients for whom it is recommended (i.e., those < 65 years of age) we were not able to wholly assess exposure rates using administrative claims data alone. Further research on guideline adherence would be valuable for this subgroup since indications for upfront chemotherapy in HR+ disease are rare [9]. It was not until the latter part of our study that mTOR and cyclin-dependent kinase (CDK) 4/6 inhibitors were introduced into clinical practice [8, 9, 44–46]. This is reflected in the low usage rates for our metastatic cohort; 4.9% and 2.1%, respectively, the latter of which is expected to increase over time.

### Resource utilization and costs

In the first Canadian study to look at health care costs specific to patients with staged HR+/HER2− BC, we have shown total annual costs to be approximately three times higher for patients with stage IV versus I–III disease ($77,112 and $22,662, respectively). Patients with stage IV disease utilized significantly more emergency, home care, and inpatient services, reflecting the known intensified care requirements in this setting. In addition, use of the NDFP was higher for patients with stage IV BC; likely a reflection of bisphosphonate and targeted therapy usage. Our data serve as a timely baseline for assessing the economic impact of new therapeutic approaches in both adjuvant (i.e., extended therapy [4]) and palliative care.

There are, of course, significant challenges associated with comparing costs between studies, due to methodological differences as well as variability in health care systems internationally and even inter-provincially [47]. However, it is important to note that while some studies confirm a similar trend of increased expense in later cancer stages [48–50], this is not always the case [51, 52], and the contributors (i.e., hospitalization, medication, etc.) can differ significantly between tumor sites [48].

## Limitations

Limitations of our study include those inherent to administrative and claims database analyses, such as missing clinical variables of interest (e.g., ethnicity, menopausal status, genetic test results, ovarian suppression rates, and recurrence or progression). Notably, lack of recurrence data restricted our ability to delineate distant recurrences from the stage I–III cohort and apply them to the stage IV cohort. Therefore, costs reported for the stage I–III cohort may be inflated. Given our province-wide, population-based sampling, biases were limited, but involved the exclusion of patients without histologic subtype (*n* = 3914) or tumor staging (*n* = 74) information and the lack of prescription drug cost data for patients under 65 years of age.

Because only publicly funded services were captured in the databases utilized, the total cost of care to society cannot be calculated (i.e., out-of-pocket patient expenses, loss of productivity). In addition, cost data in a matched control group without BC were not assessed; thus, the total costs reported for these patients represents all publicly funded health care costs and not solely HR+/HER2− BC-attributable costs.

Finally, assessing clinical outcomes or the statistical significance of differences between groups by stage at diagnosis was beyond the scope of this study, but could have provided additional context and/or highlighted implications of various treatment patterns.

## Conclusion

In our subset of patients with HR+/HER2− BC, rates of endocrine therapy appear to be lower than other similar real-world cohorts. We postulate that some patients may be incurring out-of-pocket treatment expenses not captured in the databases surveyed. Endocrine treatment is the cornerstone of therapy for patients with HR+/HER2− BC and is a critical to minimizing recurrence and prolonging survival. Hence, we must ensure optimal exposure to endocrine treatments in addition to the pursuit of new therapeutic approaches. Finally, despite more favorable survival for patients with metastatic HR+/HER- BC compared with other molecular subtypes [53], our resource utilization data indicate high inpatient treatment rates and reflect the well-known burden of illness borne by these patients.

## Electronic supplementary material

Below is the link to the electronic supplementary material.

Supplementary material 1 (DOCX 174 kb)

## References

[1] Viale G (2012). The current state of breast cancer classification. Ann Oncol.

[2] Waks AG, Winer EP (2019). Breast cancer treatment: a review. JAMA.

[3] Howlader N, Cronin KA, Kurian AW, Andridge R (2018). Differences in breast cancer survival by molecular subtypes in the United States. Cancer Epidemiol Biomark Prev.

[4] Burstein HJ, Lacchetti C, Anderson H (2019). Adjuvant endocrine therapy for women with hormone receptor-positive breast cancer: ASCO clinical practice guideline focused update. J Clin Oncol.

[5] Henry NL, Somerfield MR, Abramson VG (2019). Role of patient and disease factors in adjuvant systemic therapy decision making for early-stage, operable breast cancer: update of the ASCO Endorsement of the Cancer Care Ontario Guideline. JCO.

[6] Partridge AH, Rumble RB, Carey LA (2014). Chemotherapy and targeted therapy for women with human epidermal growth factor receptor 2–negative (or unknown) advanced breast cancer: American Society of Clinical Oncology Clinical Practice Guideline. JCO.

[7] Başaran GA, Twelves C, Diéras V (2018). Ongoing unmet needs in treating estrogen receptor-positive/HER2-negative metastatic breast cancer. Cancer Treat Rev.

[8] Almstedt K, Schmidt M (2015). Targeted therapies overcoming endocrine resistance in hormone receptor-positive breast cancer. Breast Care (Basel).

[9] Rugo HS, Rumble RB, Macrae E (2016). Endocrine therapy for hormone receptor-positive metastatic breast cancer: American Society of clinical oncology guideline. JCO.

[10] Mittmann N, Porter JM, Rangrej J (2014). Health system costs for stage-specific breast cancer: a population-based approach. Curr Oncol.

[11] World Health Organization (2004). ICD-10: international statistical classification of diseases and related health problems: tenth revision.

[12] Nofech-Mozes S, Vella ET, Dhesy-Thind SK, Hanna WP (2011). The expert panel on hormone receptor testing in breast cancer.

[13] Wolff AC, Hammond MEH, Hicks DG (2014). Recommendations for Human epidermal growth factor receptor 2 testing in breast cancer: American Society of Clinical Oncology/College of American Pathologists Clinical Practice Guideline Update. Arch Pathol Lab Med.

[14] Wolff AC, Hammond MEH, Schwartz JN (2006). American Society of Clinical Oncology/College of American Pathologists guideline recommendations for human epidermal growth factor receptor 2 testing in breast cancer. J Clin Oncol.

[15] Munro A, Alasia A, Bollman RD, Statistics Canada (2011). Self-contained labour areas: A proposed delineation and classification by degree of rurality.

[16] Charlson ME, Pompei P, Ales KL, MacKenzie CR (1987). A new method of classifying prognostic comorbidity in longitudinal studies: development and validation. J Chronic Dis.

[17] Government of Canada SC (2013) 8. Health System Indicators (Canadian Institute for Health Information – CIHI). https://www150.statcan.gc.ca/n1/pub/82-221-x/2013001/quality-qualite/qua8-eng.htm. Accessed 18 Sep 2019

[18] Cancer Care Ontario Board (2005) Guidelines for staging patients with cancer. https://stg.cancercareontario.ca/sites/ccocancercare/files/assets/CCOCancerPatientStagingGuidelines.pdf. Accessed 11 Jul 2019

[19] Elston CW (2005). Classification and grading of invasive breast carcinoma. Verh Dtsch Ges Pathol.

[20] Bank of Canada Inflation Calculator. https://www.bankofcanada.ca/rates/related/inflation-calculator/. Accessed 16 July 2019

[21] Kuwornu P, Calzavara A, Wodchis W (2017). Allocation of hospital-based outpatient clinic visit costs to individual patients.

[22] Government of Canada SC (2019) Number and rates of new cases of breast cancer by province, age, group, and sex. https://www150.statcan.gc.ca/t1/tbl1/en/tv.action?pid=1310011101. Accessed 18 Sep 2019

[23] Bastiaannet E, Charman J, Johannesen TB (2018). A European, observational study of endocrine therapy administration in patients with an initial diagnosis of hormone receptor-positive advanced breast cancer. Clin Breast Cancer.

[24] Carey LA, Perou CM, Livasy CA (2006). Race, breast cancer subtypes, and survival in the Carolina Breast Cancer Study. JAMA.

[25] Cordeiro E, Dixon M, Coburn N, Holloway CMB (2015). A patient-centered approach to wait times in the surgical management of Breast Cancer in the Province of Ontario. Ann Surg Oncol.

[26] Lizarraga I, Schroeder MC, Weigel RJ, Thomas A (2015). Surgical management of breast cancer in 2010-2011 SEER registries by hormone and HER2 receptor status. Ann Surg Oncol.

[27] Holleczek B, Stegmaier C, Radosa JC (2019). Risk of loco-regional recurrence and distant metastases of patients with invasive breast cancer up to ten years after diagnosis - results from a registry-based study from Germany. BMC Cancer.

[28] Sparano JA, Wang M, Zhao F (2012). Obesity at diagnosis is associated with inferior outcomes in hormone receptor-positive operable breast cancer. Cancer.

[29] Kim S, Park HS, Kim JY (2016). Comparisons of oncologic outcomes between triple-negative breast cancer (TNBC) and non-TNBC among patients treated with breast-conserving therapy. Yonsei Med J.

[30] Eisen A, Fletcher GG, Gandhi S (2015). Optimal systemic therapy for early breast cancer in women: a clinical practice guideline. Curr Oncol.

[31] Burstein HJ, Prestrud AA, Seidenfeld J (2010). American Society of Clinical Oncology clinical practice guideline: update on adjuvant endocrine therapy for women with hormone receptor-positive breast cancer. J Clin Oncol.

[32] Burstein HJ, Temin S, Anderson H (2014). Adjuvant endocrine therapy for women with hormone receptor-positive breast cancer: american society of clinical oncology clinical practice guideline focused update. J Clin Oncol.

[33] Livaudais JC, Lacroix A, Chlebowski RT (2013). Racial/ethnic differences in use and duration of adjuvant hormonal therapy for breast cancer in the women’s health initiative. Cancer Epidemiol Biomark Prev.

[34] Wheeler SB, Kohler RE, Reeder-Hayes KE (2014). Endocrine therapy initiation among Medicaid-insured breast cancer survivors with hormone receptor-positive tumors. J Cancer Surviv.

[35] Farias AJ, Du XL (2016). Ethnic differences in initiation and timing of adjuvant endocrine therapy among older women with hormone receptor-positive breast cancer enrolled in Medicare Part D. Med Oncol.

[36] Murphy CC, Tiro JA, Jean GW (2017). High Initiation of adjuvant hormonal therapy among uninsured stages I–III breast cancer patients treated in a safety-net healthcare system. J Womens Health (Larchmt).

[37] Daly B, Olopade OI, Hou N (2017). Evaluation of the quality of adjuvant endocrine therapy delivery for breast cancer care in the United States. JAMA Oncol.

[38] Inwald EC, Koller M, Klinkhammer-Schalke M (2015). Adjuvant endocrine therapy in pre- versus postmenopausal patients with steroid hormone receptor-positive breast cancer: results from a large population-based cohort of a cancer registry. J Cancer Res Clin Oncol.

[39] Reeder-Hayes KE, Meyer AM, Dusetzina SB (2014). Racial disparities in initiation of adjuvant endocrine therapy of early breast cancer. Breast Cancer Res Treat.

[40] Blanchette PS, Lam M, Richard L (2019). Factors associated with endocrine therapy adherence among post-menopausal women treated for early-stage breast cancer in Ontario.

[41] Farias AJ, Du XL (2017). Racial differences in adjuvant endocrine therapy use and discontinuation in association with mortality among medicare breast cancer patients by receptor status. Cancer Epidemiol Biomark Prev.

[42] Early Breast Cancer Trialists’ Collaborative Group (EBCTCG) (2005). Effects of chemotherapy and hormonal therapy for early breast cancer on recurrence and 15-year survival: an overview of the randomised trials. Lancet.

[43] Jacquet E, Lardy-Cléaud A, Pistilli B (2018). Endocrine therapy or chemotherapy as first-line therapy in hormone receptor-positive HER2-negative metastatic breast cancer patients. Eur J Cancer.

[44] Bardia A, Hurvitz S (2018). Targeted therapy for premenopausal women with HR+ , HER2− advanced breast cancer: focus on special considerations and latest advances. Clin Cancer Res.

[45] Abraham J, Coleman R, Elias A (2018). Use of cyclin-dependent kinase (CDK) 4/6 inhibitors for hormone receptor-positive, human epidermal growth factor receptor 2-negative, metastatic breast cancer: a roundtable discussion by The Breast Cancer Therapy Expert Group (BCTEG). Breast Cancer Res Treat.

[46] Messina C, Cattrini C, Buzzatti G (2018). CDK4/6 inhibitors in advanced hormone receptor-positive/HER2-negative breast cancer: a systematic review and meta-analysis of randomized trials. Breast Cancer Res Treat.

[47] Lipscomb J, Yabroff KR, Hornbrook MC (2013). Comparing cancer care, outcomes, and costs across health systems: charting the course. J Natl Cancer Inst Monogr.

[48] Seung SJ, Hurry M, Hassan S, Walton RN, Evans WK (2019). Cost-of-illness study for non-small-cell lung cancer using real-world data. Curr Oncol.

[49] Krahn MD, Zagorski B, Laporte A (2010). Healthcare costs associated with prostate cancer: estimates from a population-based study. BJU Int..

[50] Mittmann N, Liu N, Cheng SY (2020). Health system costs for cancer medications and radiation treatment in Ontario for the 4 most common cancers: a retrospective cohort study. CMAJ Open.

[51] Aly A, Johnson C, Doleh Y (2020). The real-world lifetime economic burden of urothelial carcinoma by stage at diagnosis. J Clin Pathw.

[52] Mariotto AB, Enewold L, Zhao J, Zeruto CA, Yabroff KR (2020). Medical care costs associated with cancer survivorship in the United States. Cancer Epidemiol Biomark Prev.

[53] Seung SJ, Traore AN, Pourmirza B (2020). A population-based analysis of breast cancer incidence & survival by subtype in Ontario women. Curr Oncol.

