# Supplementary material for: A population-based comparison of treatment patterns, resource utilization, and costs by cancer stage for Ontario patients with hormone receptor-positive/HER2-negative breast cancer
Source: Breast Cancer Res Treat. 2020 Oct 16;185(2):507–15. doi: 10.1007/s10549-020-05960-4 (PMC7867554; doi:10.1007/s10549-020-05960-4)
Supplement: Supplementary file 1 — Supplementary material 1 (DOCX 174 kb) [file 10549_2020_5960_MOESM1_ESM.docx]

**SUPPLEMENTARY INFORMATION**

**for**

***A population-based comparison of treatment patterns, resource utilization and costs by cancer stage for Ontario patients with hormone receptor-positive/HER2-negative breast cancer***

***Breast Cancer Research and Treatment***

Christine Brezden-Masley MD, PhD, FRCPC^1^ | Kelly E. Fathers PhD^2^ | Megan E. Coombes MSc^3^ | Behin Pourmirza MD^2^ | Cloris Xue MSc^2^ | Katarzyna J. Jerzak MD, MSc, FRCPC^4^

^1^Division of Medical Oncology and Hematology, Faculty of Medicine, University of Toronto, Mount Sinai Hospital (Toronto, Ontario, Canada)

^2^Department of Medical Affairs, Hoffmann-La Roche Limited (Mississauga, Ontario, Canada)

^3^Market Access and Pricing Department, Hoffmann-La Roche Limited (Mississauga, Ontario, Canada)

^4^Division of Medical Oncology and Hematology, Faculty of Medicine, University of Toronto, Sunnybrook Odette Cancer Center (Toronto, Ontario, Canada); katarzyna.jerzak@sunnybrook.ca

**Fig. S1** CONSORT diagram of exclusions to arrive at final cohort of female cases of HR+/HER2- breast cancer from the ICES database

Breast cancer diagnosed between Apr 1 2012 and Mar 31 2016

N=40,929

Exclude: concurrent cancer diagnosis

N=92

Remaining cases

N=40,837

Exclude: previous cancer diagnosis

N=6076

Remaining cases

N=34,761

Exclude: unknown sex

N=0

Remaining cases

N=34,761

Exclude: age <18 or >105

N=252

Remaining cases

N=34,509

Exclude: death date before diagnosis

N=8

Remaining cases

N=34,501

Exclude: malignant lymphomas

N=161

Remaining cases

N=34,340

Exclude: histologic subtype unknown

N=3,914

Remaining cases

N=30,426

Exclude: HER2 positive

N=4,902

Remaining cases

N=25,524

Exclude: ER and PR negative

N=3,277

Remaining cases

N=22,247

Exclude: AJCC stage unknown

N=74

**Final Study Cohort**

**N=22,173**

Abbreviations: AJCC, American Joint Committee on Cancer; ER, estrogen receptor; HER2, human epidermal growth factor receptor 2; IKN, encrypted ICES key number; PR, progesterone receptor.

**Table S1** List and description of datasets available to ICES

| **Dataset** | **Related Resource** | **Description** |
| --- | --- | --- |
| **Owner: Cancer Care Ontario** | | |
| Cancer Activity Level Reporting (ALR) *2005* | - Chemotherapy - Supportive drugs  - Radiation therapy | Contains patient level activity within the cancer system focused on radiation and systemic therapy services and outpatient oncology clinic visits. |
| **Owner: Ontario Ministry of Health and Long term Care** | | |
| Client Agency Program Enrolment (CAPE) *1999* | - Capitation costs | Registry of patients enrolled in a primary care model. Data elements include program type (family health team, family health organization, family health network, etc.) and patient enrolment status. |
| Home Care Database (HCD), Ontario Home Care Administrative System (OHCAS) *1990* | - Home care services | Captures information on all services provided or coordinated by Ontario Community Care Access Centres, including client data, intake and assessment information, admission and discharge, etc. |
| New Drug Funding Program (NDFP) *1995* | - Cancer drugs | Administered by Cancer Care Ontario, the NDFP funds new, and often very expensive, cancer drugs. |
| Ontario Cancer Registry (OCR) *1964* | - Cancer diagnosis | Contains the diagnosis code for invasive cancer (International Classification of Diseases version 10) and the date of diagnosis for all residents of Ontario. |
| Ontario Drug Benefit (ODB) claims *1990* | - Medication use | Contains claims for oral prescription drugs covered under the ODB program. Primarily includes drug claims for individuals 65 years of age and older, but also coverage under special ODB programs. |
| Ontario Health Insurance Plan (OHIP) claims database *1991* | - Outpatient physician visits - Laboratory services - Non-physician services  - Physician services (including costs related to breast reconstruction) | Contains claims paid by OHIP, the universal, single-payer provincial health insurance plan, for services provided by all eligible health care providers, including physicians (primary and specialist), groups, and laboratories. |
| Registered Persons Database (RPDB) *1991* | - Health service subscriber data | Contains birth and death dates, age, sex, and date of last contact with health care services in Ontario. |
| **Owner: Canadian Institute for Health Information** | | |
| Continuing Care Reporting System (CCRS) *1996* | - Complex continuing care (CCC) - Long-term care (LTC) | Contains information about residents receiving facility-based continuing care services. Range of services includes CCC, extended or chronic care, and residential care providing nursing services (that is, long-term care). |
| Discharge Abstract Database (DAD) *1988* | - Inpatient hospitalizations | Contains demographic, clinical, and administrative data for inpatient hospital admissions (patient separations). |
| National Ambulatory Care Reporting System (NACRS) *2000/2003* | - Ambulatory emergency department visit, dialysis clinic visits, and cancer clinic visits | Contains data from hospital- and community-based ambulatory care services, including same day surgery, outpatient clinics, and emergency departments. |
| National Rehabilitation Reporting System (NRS) *2000* | - Rehabilitation admissions | Contains client data from adult inpatient rehabilitation facilities, such as administrative data (referral, admission, and discharge) and health and functional characteristics. |
| Ontario Mental Health Reporting System (OMHRS) *2005* | - Mental health admissions | Contains data on patients in adult designated inpatient mental health beds in acute and psychiatric facilities. Data elements include admission and discharge dates, diagnoses, service utilization, etc. |

Reference: ICES Data Dictionary [Internet]. 2019. Available from: https://datadictionary.ices.on.ca/Applications/DataDictionary/Default.aspx

| **Table S2** Treatments and wait times among patients with stage I-III HR+ breast cancer (Ontario, 2012–2017) | | | | | | | |
| --- | --- | --- | --- | --- | --- | --- | --- |
|  | **Variable** | **Value** |  | **Stage I-III with surgery (n=20,510)** |  | **Stage I-III without surgery (n=1,499)** |  |
|  | **Surgery** |  |  |  |  |  |  |
|  | No. of surgeries within 1 year of dx | Mean (SD) |  | 1.17 (0.42) |  | NA |  |
|  | No. of days between dx and surgery | Mean (SD) |  | 48 (46) |  | NA |  |
|  |  | Median (IQR) |  | 36 (25–51) |  | NA |  |
|  | **Systemic Therapy** |  |  |  |  |  |  |
|  | Patients who received | No. (%) |  |  |  | 696 (81.9) |  |
|  | Days between dx and first tx | Mean (SD) |  |  |  | 100 (191) |  |
|  |  | Median (IQR) |  |  |  | 44 (27–83) |  |
|  | **Radiation** |  |  |  |  |  |  |
|  | Patients who received | No. (%) |  | 15,673 (76.4) |  | 261 (30.7) |  |
|  | Days between dx and first tx | Mean (SD) |  | 145 (98) |  | 286 (290) |  |
|  |  | Median (IQR) |  | 117 (83–195) |  | 187 (85–412) |  |
| Abbreviations: dx, diagnosis; HR, hormone receptor; IQR, interquartile range; NA, not applicable; tx, treatment. | | | | | | | |

| **Table S3** Treatments and wait times among patients with stage IV HR+ cancer (Ontario, 2012–2017) | | | | | | | |
| --- | --- | --- | --- | --- | --- | --- | --- |
|  | **Variable** | **Value** |  | **Stage IV**  **with surgery**  **(n=164)** |  | **Stage IV without surgery (n=649)** |  |
|  | **Surgery** |  |  |  |  |  |  |
|  | No. of surgeries within one year of dx | Mean (SD) |  | 1.08 (0.27) |  | NA |  |
|  | No. of days between dx and surgery | Mean (SD) |  | 85 (85) |  | NA |  |
|  |  | Median (IQR) |  | 43 (26, 152) |  | NA |  |
|  | Patients who received surgery only | No. (%) |  | 3 (1.8)*^a^* |  | NA |  |
|  | **Systemic Therapy** |  |  |  |  |  |  |
|  | Patients who received systemic tx | No. (%) |  | 155 (94.5)  50 (30.5)*^b^* |  | 603 (92.9) |  |
|  | Days between dx and first tx | Mean (SD) |  | 36 (27)*^b^* |  | 77 (141) |  |
|  |  | Median (IQR) |  | 31 (20–46)*^b^* |  | 35 (23–62) |  |
|  | Days between first tx and surgery | Mean (SD) |  | 161 (55)*^b^* |  | NA |  |
|  |  | Median (IQR) |  | 154 (139–198)*^b^* |  | NA |  |
|  | **Radiation** |  |  |  |  |  |  |
|  | Patients who received | No. (%) |  | 105 (64.0) |  | 370 (57.0) |  |
|  | Days between dx and first tx | Mean (SD) |  | 263 (271) |  | 201 (305) |  |
|  |  | Median (IQR) |  | 204 (91–281) |  | 44 (18–281) |  |
| *^a^*mid-point of suppressed data range, n=$\pm$2.  *^b^*in those receiving systemic tx before surgery.  Abbreviations: dx, diagnosis; HR, hormone receptor; IQR, interquartile range; tx, treatment. | | | | | | | |

| **Table S4** Number of visits, length of stay, and cost per person per year (mean ± SD) for each health care resource, by stage in patients with HR+/HER2- breast cancer (Ontario, 2012-2017) | | | | | | | | | | |
| --- | --- | --- | --- | --- | --- | --- | --- | --- | --- | --- |
|  |  |  | **Full sub cohort** | | |  | **Population utilizing** | | |  |
|  | **Resource** |  | **Stage I-III**  **(n=21,360)** |  | **Stage IV**  **(n=813)** |  | **Stage I-III** |  | **Stage IV** |  |
|  | **Professional (OHIP)** | | |  |  |  | **n=21,356***^a^* **(100%)** |  | **n=810***^a^* **(100%)** |  |
|  | Visits (no.) |  | 34.2 ± 30.8 |  | 105.8 ± 125.3 |  | 34.2 ± 30.8 |  | 105.9 ± 125.3 |  |
|  | Cost |  | $4,617 ± 3,818 |  | $11,990 ± 17,342 |  | $4,618 ± 3,818 |  | $12,005 ± 17,347 |  |
|  | **Lab (OHIP)** |  |  |  |  |  | **n=20,418 (96%)** |  | **n=672 (83%)** |  |
|  | Visits (no.) |  | 19.7 ± 19.9 |  | 21.9 ± 46.6 |  | 20.6 ± 19.9 |  | 26.4 ± 50.1 |  |
|  | Cost |  | $161 ± 151 |  | $188 ± 336 |  | $168 ± 151 |  | $228 ± 357 |  |
|  | **Inpatient (Hosp.)** | | |  |  |  | **n=9,574 (45%)** |  | **n=602 (74%)** |  |
|  | Visits (no.) |  | 0.4 ± 0.9 |  | 1.9 ± 4.2 |  | 0.8 ± 1.2 |  | 2.6 ± 4.7 |  |
|  | LOS (days) |  | 2.0 ± 11.4 |  | 24.2 ± 59.5 |  | 4.6 ± 16.7 |  | 32.7 ± 67.2 |  |
|  | Cost |  | $3,180 ± 18,194 |  | $28,711 ± 80,132 |  | $7,094 ± 26,660 |  | $38,774 ± 91,020 |  |
|  | **Inpatient (Rehab.)** | | |  |  |  | **n=354 (2%)** |  | **n=40 (5%)** |  |
|  | Visits (no.) |  | 0.0 ± 0.1 |  | 0.0 ± 0.2 |  | 0.5 ± 0.5 |  | 0.7 ± 0.8 |  |
|  | LOS (days) |  | 0.2 ± 1.9 |  | 0.8 ± 6.1 |  | 10.2 ± 10.2 |  | 15.7 ± 22.9 |  |
|  | Cost |  | $131 ± 1,589 |  | $623 ± 4,804 |  | $7,882 ± 9,570 |  | $12,654 ± 18,012 |  |
|  | **Inpatient (MH)** |  |  |  |  |  | **n=107***^a^* **(<1%)** |  | **n=3***^a^* **(<1%)** |  |
|  | Visits (no.) |  | 0.0 ± 0.1 |  | 0.0 ± 0.1 |  | 0.7 ± 0.7 |  | 0.7 ± 0.2 |  |
|  | LOS (days) |  | 0.2 ± 5.2 |  | 0.2 ± 4.1 |  | 31.7 ± 66.7 |  | 37.2 ± 40.9 |  |
|  | Cost |  | $112 ± 3,697 |  | $151 ± 2,721 |  | $22,770 ± 47,817 |  | $24,564 ± 27,457 |  |
|  | **Same Day Surgery** | | |  |  |  | **n=18,596 (87%)** |  | **n=276 (34%)** |  |
|  | Visits (no.) |  | 0.6 ± 0.9 |  | 0.3 ± 0.7 |  | 0.7 ± 0.9 |  | 0.8 ± 1.0 |  |
|  | Cost |  | $1,496 ± 1,431 |  | $498 ± 1,233 |  | $1,718 ± 1,403 |  | $1,466 ± 1,751 |  |
|  | **Hospital Outpatient** | | |  |  |  | **n=21,144 (99%)** |  | **n=803 (99%)** |  |
|  | Visits (no.) |  | 5.4 ± 4.9 |  | 11.6 ± 18.1 |  | 5.4 ± 4.9 |  | 11.7 ± 18.2 |  |
|  | Cost |  | $1,840 ± 1,669 |  | $4,040 ± 6,470 |  | $1,859 ± 1,667 |  | $4,090 ± 6,494 |  |
|  | **Home Care** |  |  |  |  |  | **n=13,148 (62%)** |  | **n=674 (83%)** |  |
|  | Cost |  | $1,013 ± 2,855 |  | $4,982 ± 8,008 |  | $1,647 ± 3,493 |  | $6,010 ± 8,438 |  |
|  | **Amb. Cancer** |  |  |  |  |  | **n=18,300 (86%)** |  | **n=713 (88%)** |  |
|  | Cost |  | $7,061 ± 6,699 |  | $15,177 ± 14,091 |  | $8,241 ± 6,531 |  | $17,305 ± 13,768 |  |
|  | **Amb. Emergency** | | |  |  |  | **n=13,277 (62%)** |  | **n=673 (83%)** |  |
|  | Cost |  | $276 ± 608 |  | $1,249 ± 2,648 |  | $446 ± 722 |  | $1,509 ± 2,843 |  |
|  | **Amb. Dialysis** |  |  |  |  |  | **n=63***^a^* **(<1%)** |  | **n=3***^a^* **(<1%)** |  |
|  | Cost |  | $109.09 ± 2,875 |  | $91 ± 2,572 |  | $36,985 ± 38,223 |  | $24,680 ± 42,125 |  |
|  | **Drug (NDFP)** |  |  |  |  |  | **n=6,805 (32%)** |  | **n=461 (57%)** |  |
|  | Cost |  | $275 ± 2,421 |  | $1,103 ± 7,662 |  | $862 ± 4,230 |  | $1,945 ± 10,099 |  |
|  | **Drug (ODB)** |  |  |  |  |  | **n=16,937 (79%)** |  | **n=687 (85%)** |  |
|  | Cost |  | $1,566 ± 3,222 |  | $3,019 ± 5,689 |  | $1,975 ± 3,504 |  | $3,573 ± 6,028 |  |
|  | **CCC** |  |  |  |  |  | **n=334 (2%)** |  | **n=82 (10%)** |  |
|  | Cost |  | $222 ± 3,745 |  | $4,291 ± 24,021 |  | $14,219 ± 26,453 |  | $42,542 ± 64,318 |  |
|  | **Long Term Care** | | |  |  |  | **n=507 (2%)** |  | **n=38 (5%)** |  |
|  | Visits (no.) |  | 0.1 ± 0.7 |  | 0.2 ± 0.9 |  | 3.9 ± 2.8 |  | 3.5 ± 2.6 |  |
|  | LOS (days) |  | 4.68 ± 37.0 |  | 7.0 ± 41.8 |  | 197.1 ± 140.9 |  | 149.0 ± 128.9 |  |
|  | Cost |  | $599 ± 4,825 |  | $999 ± 6,323 |  | $25,252 ± 18,943 |  | $21,376 ± 20,738 |  |
| *^a^*mid-point of suppressed data range, n=$\pm$2.  Abbreviations: Amb., ambulatory; CCC, Complex Continuing Care; HR, hormone receptor; Hosp., hospital; LOS, length of stay; LTC, MH, Mental Health; NDFP, New Drug Funding Program; ODB, Ontario Drug Benefit; OHIP, Ontario Health Insurance Plan; Rehab., rehabilitation; SDS, Same Day Surgery. | | | | | | | | | | |
